# Nitrogen-Doped Graphene Quantum Dot-Passivated δ-Phase CsPbI_3_: A Water-Stable Photocatalytic Adjuvant to Degrade Rhodamine B

**DOI:** 10.3390/molecules28217310

**Published:** 2023-10-28

**Authors:** Yiting Gu, Xin Du, Feng Hua, Jianfeng Wen, Ming Li, Tao Tang

**Affiliations:** College of Science & Key Laboratory of Low-Dimensional Structural Physics and Application, Education Department of Guangxi Zhuang Autonomous Region, Guilin University of Technology, Guilin 541004, China; yiting10596@163.com (Y.G.); duxin0727@126.com (X.D.); huafeng6026@163.com (F.H.); wjfculater@163.com (J.W.)

**Keywords:** nitrogen doping, graphene quantum dot, perovskite, photodegradation

## Abstract

Inorganic halide perovskite CsPbI_3_ is highly promising in the photocatalytic field for its strong absorption of UV and visible light. Among the crystal phases of CsPbI_3_, the δ-phase as the most aqueous stability; however, directly using it in water is still not applicable, thus limiting its dye photodegradation applications in aqueous solutions. Via adopting nitrogen-doped graphene quantum dots (NGQDs) as surfactants to prepare δ-phase CsPbI_3_ nanocrystals, we obtained a water-stable material, NGQDs-CsPbI_3_. Such a material can be well dispersed in water for a month without obvious deterioration. High-resolution transmission electron microscopy and X-ray diffractometer characterizations showed that NGQDs-CsPbI_3_ is also a δ-phase CsPbI_3_ after NGQD coating. The ultraviolet-visible absorption spectra indicated that compared to δ-CsPbI_3_, NGQDs-CsPbI_3_ has an obvious absorption enhancement of visible light, especially near the wavelength around 521 nm. The good dispersity and improved visible-light absorption of NGQDs-CsPbI_3_ benefit their aqueous photocatalytic applications. NGQDs-CsPbI_3_ alone can photodegrade 67% rhodamine B (RhB) in water, while after compositing with TiO_2_, NGQDs-CsPbI_3_/TiO_2_ exhibits excellent visible-light photocatalytic ability, namely, it photodegraded 96% RhB in 4 h. The strong absorption of NGQDs-CsPbI_3_ in the visible region and effective transfer of photogenerated carriers from NGQDs-CsPbI_3_ to TiO_2_ play the key roles in dye photodegradation. We highlight NGQDs-CsPbI_3_ as a water-stable halide perovskite material and effective photocatalytic adjuvant.

## 1. Introduction

In recent years, with the rapid development of the economy and technology, organic dyes have been widely used and discharged into industrial wastewater. Especially, rhodamine B (RhB), which is widely used for dyeing fabrics, paints, acrylics, and biological products, has become abundant in wastewater and highly toxic to organisms [1]. Previous studies have shown that RhB can induce growth retardation and liver damage, erythrocyte hemolysis, and suppression of the immune response in isolated spleen cells [2]. Other studies have suggested that RhB is mutagenic and carcinogenic [3] and could produce local sarcomas [2]. Therefore, it is urgent to develop economic and effective ways to remove RhB in wastewater. Producing highly oxidative active species is the basic technique for removing organic pollutants. Photocatalysis is a feasible and economic way to produce oxidative species and has been widely studied [4,5,6]. Fujishima and Honda first reported the photocatalytic performance of TiO_2_ [7], and since then many scholars have investigated its photodegradation effect [8,9]. However, its wide band gap of 3.2 eV determines that only UV light can be absorbed, and visible light, the main component of sunlight, cannot be well captured by TiO_2_ [10]. To obtain a visible-light photocatalyst, one way is to choose a photocatalytic material with a narrower band gap. Typically, graphitic C_3_N_4_, with a band gap of ~2.71 eV, can absorb visible light and has been proven effective in visible-light photodegradation [11], but the high carrier recombination rate remarkably restrains its photocatalytic performance [12]. The other way is compositing a visible-light responsive photocatalytic adjuvant with TiO_2_, which turns visible light into photogenerated carriers and effectively transfers them to TiO_2_, and then charged oxidative species are produced to degrade organic dyes. Carbon-based materials are universal adjuvants due to their widely tunable bandgaps, ease of composition, good aqueous dispersibility, and availability of carrier separation and transfer [13,14,15,16]. However, carbon-based materials suffer from weak visible-light absorption ability because their absorption mainly comes from the energy transition from σ or π to π* orbitals [17]. Searching for an alternative adjuvant with strong absorption in the visible region is beneficial to improving the visible-light photocatalytic performance of TiO_2_.

Halide perovskite, the rising star in optoelectronics, has been successfully applied in photocatalytic fields such as carbon dioxide reduction [18], water splitting [19], and dye removal [20]. Its strong visible-light acquisition ability plays the key role in photocatalysis. However, poor water stability generally is the Archilles heel of halide perovskite, so such experiments have to be conducted in organic solvents or after water-resistant coating [18,19,20]. With the invasion of water, the perovskite structure tends to stretch, distort, and tilt, turning α-, β-, and γ-phases into δ-phase [21]. δ-phase halide perovskite is generally deemed as a waste phase because it no longer exhibits typical perovskite characteristics, for example, strong photoluminescence (PL) and high quantum yield (QY) [22,23]. Also, because of this, δ-phase perovskite is the most water stable, and the low PLQY means very few photogenerated carriers undergo direct recombination. Both of these properties are very suitable for aqueous photocatalysis.

Herein, we studied the photodegradation performance of δ-CsPbI_3_ nanocrystals on RhB. The δ-CsPbI_3_ nanocrystals synthesized by the conventional method using oleic acid and oleylamine as surfactants tend to aggregate and cannot be well dispersed in water, so it is hard to perform photodegradation experiments. By using nitrogen-doped graphene quantum dots (NGQDs) as surfactants to synthesize δ-phase CsPbI_3_ [24], we obtained NGQDs-CsPbI_3_, which could be perfectly dispersed and stably stored in water for a month. After compositing with TiO_2_, under visible light, the photogenerated electrons in NGQDs-CsPbI_3_ transfer to TiO_2_, producing the oxidant radicals •O_2_^−^ and •OH, and the photogenerated holes left in NGQDs-CsPbI_3_ combine with H_2_O/OH^−^ to produce •OH, finally oxidizing RhB into mineralization products. NGQDs-CsPbI_3_/TiO_2_ exhibits excellent visible-light photocatalytic ability, which could photodegrade 96% RhB in 4 h. As a water-stable material, NGQDs-CsPbI_3_ shows bright prospects in photocatalysis, and it also opens the door to resurrecting the potential of δ-phase halide perovskites.

## 2. Results

**Figure 1:**
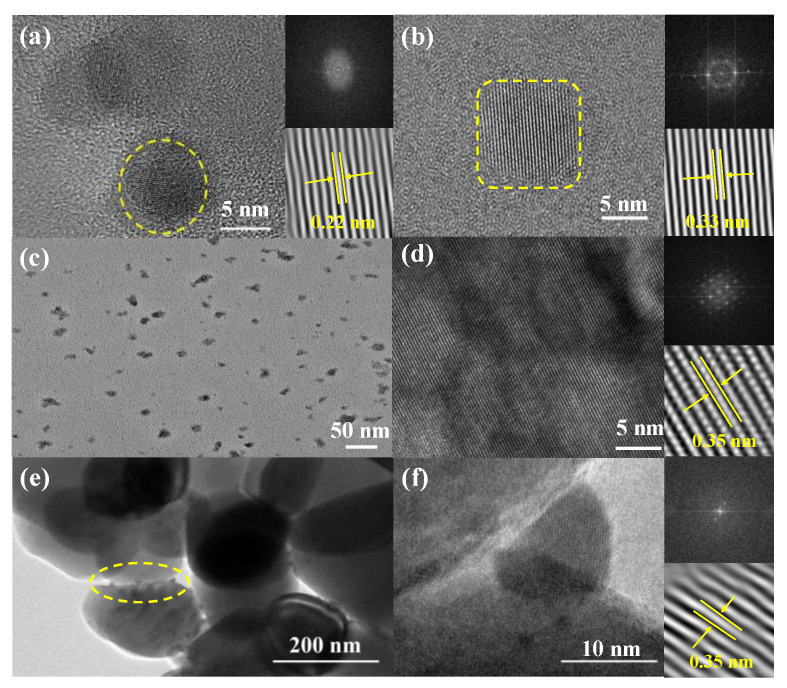
High-resolution TEM images of (**a**) NGQDs and (**b**) δ-CsPbI_3_. (**c**) Large-scale and (**d**) high-resolution TEM images of NGQDs-CsPbI_3_. (**e**) Large-scale and (**f**) high-resolution TEM images of NGQDs-CsPbI_3_/TiO_2_. The lattice diagrams are the corresponding SAED patterns.

Typical TEM images of the synthesized samples are shown in Figure 1. The NGQDs are sized 1–10 nm [Figure S1a], with 65.09, 24.05, and 10.76 at.% of C, O, and N, respectively [Figure S1b]. Figure 1a shows a single NGQD of ~5 nm with a lattice stripe distance of ~0.22 nm, which is similar to that of graphite $\left(11\bar{2}0\right)$ facets [25]. The δ-phase CsPbI_3_ nanocrystals are sized 5–20 nm [Figure S2a]. A typical 5 nm nanocrystal is shown in Figure 1b, and the lattice stripes are clearly resolved with interplanar spacing of 0.33 nm, corresponding to (212) planes of orthorhombic δ-CsPbI_3_ [26]. The borders of NGQDs-CsPbI_3_ crystals are extremely irregular [Figure 1c], and their sizes are observably enlarged, obviously ascribed to the outcome of adopting NGQDs as surfactants. Figure 1d shows a large-size NGQDs-CsPbI_3_ microcrystal, and its atomic distribution can be clearly seen with interplanar spacing of 0.35 nm. Figure S2c,d shows two other NGQDs-CsPbI_3_ nano- or microcrystals, for which different crystal spacings are exhibited. One possibility is that these spacings correspond to different crystal planes of δ-CsPbI_3_, and another is that the crystal structures of NGQDs-CsPbI_3_ are miscellaneous since nonstoichiometric NGQDs might bring about different strains to build δ-CsPbI_3_ with different crystal constants [27]. Among the large particles of TiO_2_, the NGQDs-CsPbI_3_ crystals can also be resolved [see the yellow circle in Figure 1e]. From the crystal plane distance of 0.35 nm, one can know it is a NGQDs-CsPbI_3_ nanocrystal [Figure 1f].

**Figure 2:**
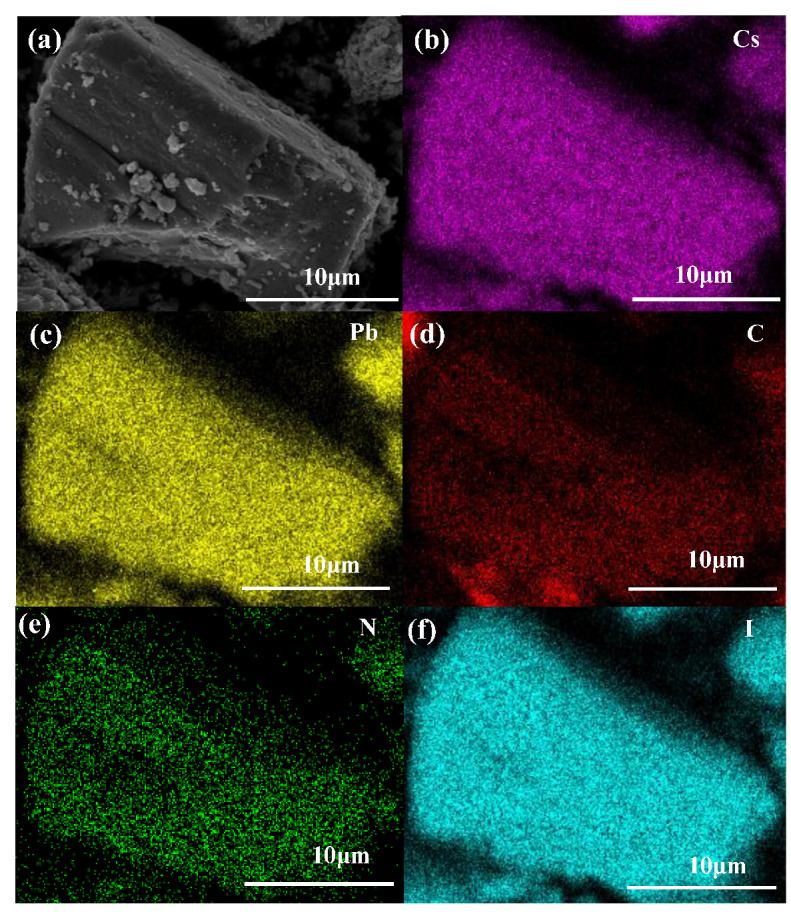
(**a**) SEM image of NGQDs-CsPbI_3_ microcrystal and EDS elemental mapping of (**b**) Cs L, (**c**) Pb L, (**d**) C K, (**e**) N K, and (**f**) I L.

The EDS elemental mapping of an NGQDs-CsPbI_3_ microcrystal is shown in Figure 2. Typical elements of NGQDs-CsPbI_3_, such as Cs, Pb, I, C, and N, can be found uniformly distributed. From Figure S3, one can know the atomic ratio of Cs:Pb:I ≈ 1:1:3, according to the formula of halide perovskite. The atomic ratio of Cs:C is ~1:8, and it is must be stressed that the NGQD portion in NGQDs-CsPbI_3_ may be overrated since NGQDs are used as surfactants and attached on the CsPbI_3_ surface.

**Figure 3:**
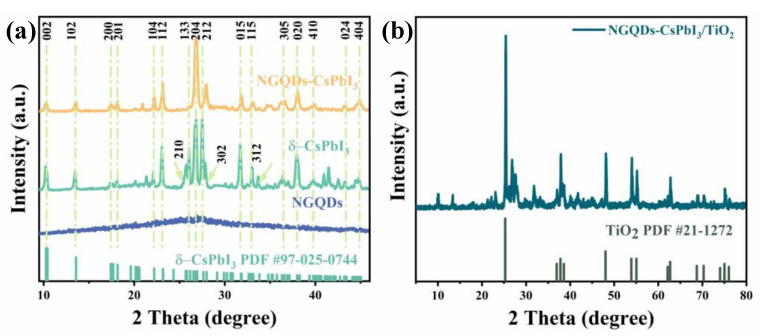
(**a**) XRD patterns of NGQDs, δ-CsPbI_3_, and NGQDs-CsPbI_3_. (**b**) XRD pattern of NGQDs-CsPbI_3_/TiO_2_.

The XRD patterns are shown in Figure 3a. The prominent peaks of δ-CsPbI_3_ are (002), (102), (200), (201), (111), (112), (210), (113), (212), and (302) at 10.27°, 13.43°, 17.42°, 18.20°, 21.41°, 23.27°, 25.70°, 26.03°, and 27.55°, respectively, which are totally in accordance with the standard PDF25-0744 of orthorhombic δ-phase CsPbI_3_ [28]. NGQDs-CsPbI_3_ exhibits similar characteristics, and in its XRD plot, the typical (002) peak of NGQDs is found at around 26° and is not well resolved [29]. Although some peaks are inconspicuous, NGQDs-CsPbI_3_ can still be reasonably seen as orthorhombic δ-phase CsPbI_3_ with NGQDs on the surface. The XRD pattern of NGQDs-CsPbI_3_/TiO_2_ [Figure 3b] demonstrates no new peaks aside from those of NGQDs-CsPbI_3_ and TiO_2_, meaning that the two parts are merely physically composited.

**Figure 4:**
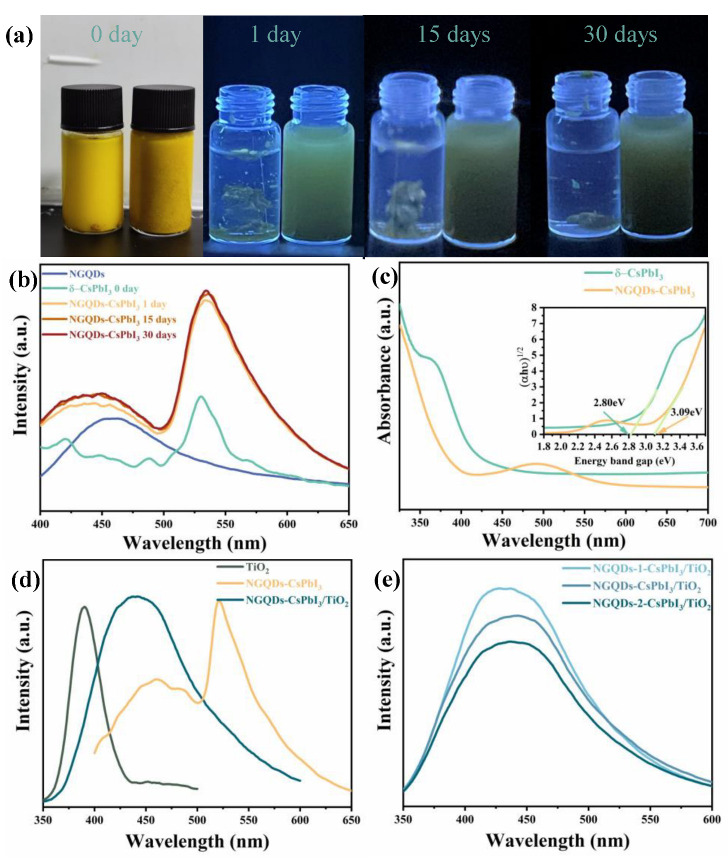
(**a**) Optical images under sun and UV light of δ-CsPbI_3_ (**left**) and NGQDs-CsPbI_3_ (**right**) nanocrystals in water. (**b**) Time-dependent PLs of NGQDs-CsPbI_3_. (**c**) The absorption spectra of δ-CsPbI_3_ and NGQDs-CsPbI_3_. (**d**) The PLs of NGQDs-CsPbI_3_ before and after compositing with TiO_2_. (**e**) The PLs of NGQDs-CsPbI_3_/TiO_2_ with different NGQD mass ratios. The excitation wavelength is 330 nm.

The aqueous stability of the photocatalyst is a key factor on deciding whether it can be used in water to photodegrade organic dyes. In Figure 4a, one can find that the newly synthesized δ-CsPbI_3_ nanocrystals can be well dispersed in water; however, shortly after preparation, they are inclined to aggregate and hard to redisperse even with stirring, so δ-CsPbI_3_ nanocrystals are hardly used as aqueous photocatalysts. On the contrary, NGQDs-CsPbI_3_ crystals could be well dispersed for a month, which is obviously highly related to the good dispersibility of NGQDs in water [30]. Here, we highlight the effect of nitrogen doping in enhancing dispersion, since we replaced NGQDs with graphene quantum dots (GQDs) to prepare GQDs-CsPbI_3_ under the same conditions and found that, totally like δ-CsPbI_3_, GQDs-CsPbI_3_ tended to aggregate quickly. In Figure 4b, after 30 days, the PL shapes and intensities of NGQDs-CsPbI_3_ nanocrystals change very slightly, demonstrating the water stability of NGQDs-CsPbI_3_. It can also be found that the PL peak of NGQDs-CsPbI_3_ can be deconvoluted into two parts: one is ascribed to NGQDs centered at 461 nm, and the other is ascribed to δ-CsPbI_3_ centered at 521 nm. The δ-CsPbI_3_ part demonstrates the typical green light of δ-phase iodide perovskite, corresponding to self-trapped exciton emission [31]. In Figure 4c, via Tauc plotting, one can find that the bandgap of NGQDs-CsPbI_3_ (3.09 eV) is a bit larger than that of δ-CsPbI_3_ (2.8 eV); however, in the visible region, especially around 521 nm, the absorption of NGQDs-CsPbI_3_ is obviously stronger. Such absorption is ascribed to the transition from the ground states to trap states, namely, passivation through NGQDs can enhance the trapping exciton absorption of δ-CsPbI_3_. The improvement in visible-light absorption undoubtedly benefits the photocatalytic ability of NGQDs-CsPbI_3_.

Figure 4d shows the PLs of TiO_2_ and NGQDs-CsPbI_3_ before and after compositing. In the PL spectrum of NGQDs-CsPbI_3_/TiO_2_, the subpeak ascribed to δ-CsPbI_3_ (centered at 521 nm) is almost quenched, and the PL of TiO_2_ (centered at 385 nm) is also invisible. NGQDs-CsPbI_3_/TiO_2_ emits light with a single peak centered at ~440 nm, representing the characteristics of NGQDs, since it only exhibits a slight blue-shift compared to NGQDs alone (~460 nm). Therefore, we can speculate that the photogenerated electrons or holes in TiO_2_ and CsPbI_3_ are effectively transferred, and some of them recombine on NGQDs, namely on the coating surface of NGQDs-CsPbI_3_ crystals. In Figure 4e, by tuning the NGQD mass ratio in NGQDs-CsPbI_3_ (the NGQD mass is 0.5, 0.7, and 0.9 mg in NGQDs-1-CsPbI_3_, NGQDs-CsPbI_3_, and NGQDs-2-CsPbI_3_, respectively, and the CsPbI_3_ mass is 72 mg), it is found that the PL positions remain invariable, merely along with sightly increased PL intensity due to the increase in NGQD weight. In a word, by compositing NGQDs-CsPbI_3_ with TiO_2_, the photoinduced carriers in both can be effectively separated and transferred, which is highly beneficial to photocatalytic applications [32].

**Figure 5:**
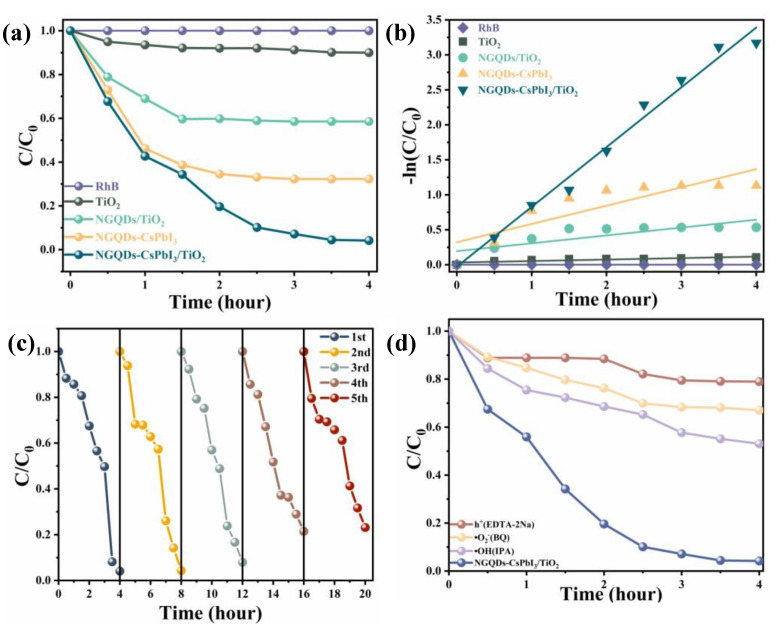
(**a**) Effects of different samples on the photocatalytic degradation of RhB under visible light (Xe lamp). (**b**) The corresponding degradation kinetic behaviors. (**c**) Photocatalytic cycle tests of NGQDs-CsPbI_3_/TiO_2_. (**d**) Effects of scavengers on the catalytic effect of NGQDs-CsPbI_3_/TiO_2_.

Taking 100 mL RhB water solution (10 mg/L) as a reference, we studied the visible-light (λ > 420 nm) photodegradation activities of NGQDs-CsPbI_3_/TiO_2_ (72.7 mg/250 mg). The effects of TiO_2_ (250 mg), NGQDs (7 mg), NGQDs/TiO_2_ (7 mg/250 mg), and NGQDs-CsPbI_3_ (72.7 mg) are also supplied for comparison. The photodegradation results are shown in Figure 5a, where C_0_ and C are the initial and real-time concentrations of RhB, respectively, and C/C_0_ is determined by the absorbance of RhB at 554 nm. Due to the bad dispersibility of δ-CsPbI_3_, it was hard to assess its photodegradation ability, so the related results are not shown here.

Obviously, the visible-light photocatalytic ability of TiO_2_ or NGQDs alone is negligible, and the RhB photodegradation ratio is enhanced after compositing [30]. It is worth noting that NGQDs-CsPbI_3_ alone displays decent photocatalytic activity, degrading 67% RhB in 4 h (see Section 4.4 for the calculation of photodegradation efficiency). The PLQY of NGQDs-CsPbI_3_ is ~3% (Table S1) and together with its broad absorption range [Figure 4c], many indirect recombined photogenerated carriers can effectively participate in the photodegradation process. To further improve the photocatalytic activity, those photocarriers must be transferred to reduce the possibility of direct recombination. By compositing NGQDs-CsPbI_3_ with TiO_2_, RhB can be nearly completely photodegraded (96%) after 4 h. Using total organic carbon (TOC) as a reference to measure the mineralization rate of RhB, similar photodegradation efficiency can be obtained, which is 94% in 4 h (Figure S4). Since the difference in photodegradation efficiency obtained by these two references (RhB absorbance and TOC) is small, all further discussion about the photodegradation of RhB is based on the former reference. The aforementioned discussion about Figure 4d shows that photocarriers are effectively transferred between TiO_2_ and NGQDs-CsPbI_3_ and TiO_2_ is almost entirely inactive to visible light; hence, effective photocarrier transfer from NGQDs-CsPbI_3_ to TiO_2_ is the reason why the photodegradation activity can be improved. The corresponding photodegradation kinetics were fitted using the first-order reaction equation:

(1) $$ln\frac{C}{C_{0}}=-kt$$

where k is the photocatalytic efficiency [33]. The k value of NGQDs-CsPbI_3_/TiO_2_ is 0.85, almost 3 times that of NGQDs-CsPbI_3_ alone [0.26, Figure 5b]. In order to further evaluate the usefulness of NGQDs-CsPbI_3_/TiO_2_ nanocrystals as photocatalytic materials, cyclic experiments of RhB photodegradation were performed [Figure 5c]. Regrettably, a slight decrease in photodegradation activity appears in the 3rd to 5th cycles, with 92%, 79%, and 77%, respectively. This is due to the mass loss of NGQDs-CsPbI_3_/TiO_2_, especially NGQDs-CsPbI_3_ nanocrystals, since it is difficult to completely collect nano-size particles in water through high-speed centrifugation. In Figure S5, one can find that using different ratios of NGQDs as surfactants to synthesize NGQDs-CsPbI_3_ changes the photodegradation ability of NGQDs-CsPbI_3_/TiO_2_, and the optimal ratio is adopted in our experiments.

Generally, photogenerated •O_2_^−^, holes (h^+^), and •OH play important roles in the degradation of organic dyes [34]. In order to identify which free radicals are dominant in photodegradation by NGQDs-CsPbI_3_/TiO_2_, radical capture experiments were conducted. As shown in Figure 5d, three scavengers, benzoquinone (BQ, 0.01 g/L), disodium ethylenediaminetetraacetate (EDTA-2Na, 0.02 g/L), and isopropyl alcohol (IPA, 0.02 g/L), were used in this study to capture the •O_2_^−^, h^+^, and •OH radicals, respectively [35]. The RhB degradation rates of NGQDs-CsPbI_3_/TiO_2_ nanocomposites were 21%, 33%, and 47% in the presence of EDTA-2Na, BQ, and IPA, respectively. The results showed that in our photocatalytic process, these three active components, •O_2_^−^, •OH, and h^+^, are all massively produced and participate in dye oxidation, and the roles of •O_2_^−^ and h^+^ are a bit more important than that of •OH. Regrettably, several capture agents (including AgNO_3_, K_2_Cr_2_O_7_, and KBrO_3_) were used to capture e^−^, and it was found that RhB was rapidly adsorbed and it was difficult to assess the photodegradation ability after e^−^ capture.

In addition, Table 1 lists a comparison of relevant photocatalysts that have been used in several studies to degrade contaminants. Under visible light, the photodegradation ability of TiO_2_ is negligible. Most halide perovskites can photodegrade dyes effectively, and these results show a good foreground of photocatalytic applications of perovskites. However, such experiments have been conducted in organic solution to ensure the stability of perovskites. As is known, the removal of organic contamination in wastewater is the first thing to be resolved; hence, to a certain degree, developing water-stable photocatalysts is more important than enhancing the photocatalytic efficiency alone. Obviously, NGQDs-CsPbI_3_/TiO_2_ is a promising photocatalyst due to its feasibility of direct use in water, although there is much room for improvement in the photocatalytic efficiency.

## 3. Discussion

**Figure 6:**
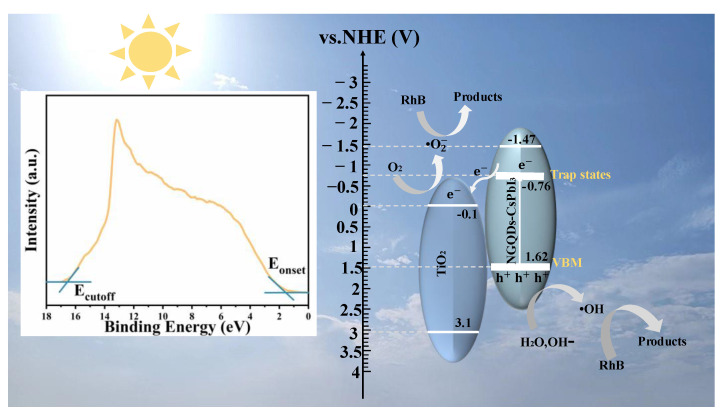
Mechanism of the photocatalytic process of NGQDs-CsPbI_3_/TiO_2_, and the inset is the UPS spectra of NGQDs-CsPbI_3_.

Figure 6 shows a schematic diagram of the RhB photodegradation process of NGQDs-CsPbI_3_/TiO_2_. Using the vacuum level as a reference, the UPS measurement [inset of Figure 6] can define the valence band maximum (VBM) by E_VBM_ = −[21.22 − (E_cutoff_ − E_onset_)]. For NGQDs-CsPbI_3_, the E_cutoff_ is 16.53 eV and the E_onset_ is 1.59 eV, so the E_VBM_ is −6.12 eV. Considering the band gap is 3.09 eV, we know that the maximum conduction band (CBM) is −3.03 eV. The emissive center is located at 521 nm, implying that the energy difference between the trap states and VBM is 2.38 eV, namely, the energy level of the trap states is −3.74 eV. Conventionally, we express energy levels using normal hydrogen electrode (NHE) as a reference [E(NHE) = −4.5 − E(vacuum), which represents the relationship between NHE and the vacuum energy levels], and the energy levels of CBM, the trap states, and VBM are −1.47, −0.76, and 1.62 eV, respectively. The photocatalytic mechanism of NGQDs-CsPbI_3_/TiO_2_ can be reasonably inferred according to the following expressions (2)–(8):

(2) $${NGQDs-CsPbI}_{3}/{TiO}_{2}+dyes\to *dyes$$

(3) $$h\nu \left({on\;NGQDs-CsPbI}_{3}\right)\to h^{+}+e^{-}$$

where *dye denotes the dye molecules activated by NGQDs-CsPbI_3_/TiO_2_. When NGQDs-CsPbI_3_/TiO_2_ is exposed to visible light, electrons and holes are only photogenerated in NGQDs-CsPbI_3_ since TiO_2_ is inactive under visible light. It also must be stressed that the electrons in NGQDs-CsPbI_3_ can only be excited from VBM to the trap states since the photon energy (λ > 420 nm) cannot compensate for the bandgap of NGQDs-CsPbI_3_ (3.09 eV). The primary function process of •OH radicals is shown as the following expressions (4)–(6):

(4) $$H_{2}O\;⇋\;H^{+}+{OH}^{-}$$

(5) $$h^{+}+{OH}^{-}\to •OH$$

(6) •OH+∗dyes→mineralization products

As shown in Figure 6, the photogenerated holes in NGQDs-CsPbI_3_ combine with OH^−^ generated by water to form •OH, and then •OH reacts with *dyes to produce mineralization products. Generally speaking, oxidizing OH^−^ into •OH requires the holes to have a high potential. For example, in TiO_2_ [39], Bi-doped LaFeO_3_ [40], and g-C_3_N_4_ [41], the hole potentials are 1.83, 1.97, and 1.84 V, respectively. However, in halide perovskite, this potential can decrease to as low as 1.1 V [36]. The VBM of NGQDs-CsPbI_3_ is 1.62 eV, which might be enough for holes to generate •OH radicals in such a perovskite material. Moreover, the broad PL and wide absorption of NGQDs-CsPbI_3_ indicate that the excited electrons and holes are distributed in a wide energy range, which means the potentials of many holes are higher than 1.62 V, further boosting the oxidation of OH^−^ into •OH to degrade pollutants. The photodegradation process of •O_2_^−^ radicals can also be seen in Figure 6, as follows:

(7) $$O_{2}+e^{-}\to {•O}_{2}^{-}$$

(8) $${•O}_{2}^{-}+*dyes\to mineralization\;products$$

The electrons produced by photoexcitation are transferred from the trap states of NGQDs-CsPbI_3_ to the CBM of TiO_2_, combine with the adsorbed O_2_ to form •O_2_^−^, and then •O_2_^−^ reacts with *dyes to form mineralized compounds. Certainly, some •O_2_^−^ can be captured by H_2_O_2_ to form •OH, and again, •OH reacts with *dyes to form mineralized products.

## 4. Materials and Methods

### 4.1. Materials

Citric acid (CA, anhydrous), urea (99.5%), cesium iodide (CsI, 99.9%), oleylamine (OLA, 96%), dimethylformamide (DMF, 99.8%), and RhB (98%) were purchased from Shanghai Aladdin Biochemical Technology Co., Ltd. (Shanghai, China). Lead iodide (PbI_2_, 99.998%) was purchased from Alfa. Oleic acid (OA, 90%), P25 TiO_2_ (80% anatase and 20% rutile), isopropyl alcohol (IPA), p-benzoquinone (BQ), and disodium EDTA-2Na were purchased from Xilong Chemical Co., Ltd. (Shantou, China). All chemicals were used directly without further purification.

### 4.2. Sample Synthesis

#### 4.2.1. Synthesis of NGQDs

First, 0.53 g CA and 0.6 g urea were dissolved in 12 mL deionized water and stirred to form a clarified solution. The solution was then transferred to a 50 mL Teflon autoclave. The sealed autoclave was heated to 160 °C in an oven and maintained for 8 h. The final product was collected by adding ethanol to the solution and centrifuging at 5000 rpm for 5 min. Finally, the sediment was dried at 60 °C to obtain NGQDs.

#### 4.2.2. Synthesis of δ-CsPbI_3_

First, 0.26 g CsI and 0.46 g PbI_2_ were dissolved in 2 mL DMF, and the solution was heated to 50 °C and held at this temperature for 20 min. Subsequently, 0.4 mL OA and 0.2 mL OLA were added to stabilize the precursor solution, and then the precursor solution was heated to 90 °C and held at this temperature for 30 min. Finally, 0.2 mL precursor solution was quickly added to 4 mL deionized water (under vigorous stirring); after drying, δ-CsPbI_3_ nanocrystals were obtained.

#### 4.2.3. Synthesis of NGQDs-CsPbI_3_

The synthesis process of NGQDs-CsPbI_3_ was similar to that of δ-CsPbI_3_, except that the organic ligands OA and OLA were replaced by NGQDs. First, 0.26 g CsI and 0.46 g PbI_2_ were dissolved in 2 mL DMF. Next, 7 mg NGQDs was added to obtain a precursor solution. Then, 0.2 mL precursor solution was rapidly added to 4 mL of deionized water (under vigorous stirring) to immediately generate NGQDs-CsPbI_3_ crystals. For comparison, the initial mass ratio of NGQDs was tuned to synthesize NGQDs-CsPbI_3_, and these were named as NGQDs-1-CsPbI_3_ and NGQDs-2-CsPbI_3_ with initial NGQD masses of 5 and 9 mg, respectively.

#### 4.2.4. Synthesis of NGQDs-CsPbI_3_/TiO_2_

First, 250 mg TiO_2_ was added into the above NGQDs-CsPbI_3_ solution (including 72.7 mg NGQDs-CsPbI_3_, and the mass of NGQDs is 0.7 mg) and uniformly mixed with stirring; after drying, NGQDs-CsPbI_3_/TiO_2_ composite was obtained.

### 4.3. Characterization

The crystal structures were characterized using a Miniflex-600 X-ray diffractometer (XRD, JEOL, Tokyo, Japan). The morphologies were assessed using a field emission transmission electron microscope (TEM, JEM2100F, Tokyo, Japan) equipped with a selected area electron diffractometer (SAED) and a scanning electron microscope (SEM, TESCAN MIRA LMS, Brno, Czech Republic) equipped with an energy dispersive spectroscope (EDS). Surface analyses were carried out with an Escalab-250XI X-ray photoelectron spectrometer (XPS) from Thermo Fisher Scientific (Waltham, MA, USA). Ultraviolet-visible (UV-vis) absorption spectra were obtained using a PerkinElmer Lambda 750. Photoluminescence (PL) spectra were determined using an Edinburgh FL/FS900 carry Eclipse (Cheadle, UK). UV photoelectron spectroscopy (UPS) measurements were performed on a photoelectron spectrometer (ESCALAB 250Xi) with a He I source of 21.22 eV.

### 4.4. Photodegradation Test

The photocatalytic activities were investigated by photodegrading RhB (100 mL, 10 mg/L) under a xenon lamp (PLS-SXE 300, 300 W, λ > 420 nm). The adsorption–desorption equilibrium of RhB and photocatalysts was achieved after being stirred in the dark for 0.5 h. Every 0.5 h, 4 mL solution was taken out to determine its RhB concentration using a Lambda 950 UV-Vis spectrophotometer. The recycle photodegradation experiments were conducted by repeatedly collecting the photocatalysts via centrifugation and drying.

The concentration of RhB was measured by UV-vis spectrophotometry and the degradation efficiency of RhB was calculated as:

(9) $$degradation\;ratio\;(\%)=\frac{C_{0}-C}{C_{0}}\times 100=\frac{A_{0}-A}{A_{0}}\times 100$$

where C_0_ and C are the initial and real-time concentrations of RhB, respectively; and A_0_ and A are the initial and real-time absorbance of RhB at 554 nm, respectively. The mineralization rate of RhB solutions was measured using a TOC analyzer (Shimadzu, TOC-L CPN, Tsushima, Japan). The photodegradation ratio was determined by the following formula:

(10) $$degradation\;ratio\;(\%)=\frac{{TOC}_{0}-TOC}{TOC_{0}}\times 100$$

where TOC_0_ and TOC are respectively the initial and real-time TOC of RhB solution.

## 5. Conclusions

Water-stable NGQDs-CsPbI_3_ halide perovskite was successfully prepared by the thermal injection method at room temperature. It exhibits typical characteristics of δ-phase CsPbI_3_, namely strong absorption in the visible region and low PL QY. Due to the coating and passivation through NGQDs, such a material can be well dispersed and is stable in water for a month, hence it can effectively photodegrade RhB. After compositing with TiO_2_, the photodegradation ability is further improved because the photogenerated e-h pairs can be effectively separated and transferred. We highlight NGQDs-CsPbI_3_ as a water-stable perovskite with great potential in the photocatalytic field.

## Figures and Tables

**Table 1:** Comparison of dye degradation effects of relevant photocatalysts under visible light.

No.	Catalysts	Dye Solution	Efficiency	Ref.
1	TiO_2_	RhB in water	10% in 4 h	[8]
2	CsPbBr_3_	RhB in toluene/ethanol	89% in 100 min	[36]
3	CsPbCl_3_	RhB in toluene/ethanol	90% in 100 min	[36]
4	Cs_3_AgInCl_3_	Sudan Red in ethanol	98.5% in 16 min	[20]
5	Cs_4_MnBiCl_12_	RhB in ethanol	97% in 7 min	[37]
6	Cs_2_AgBiBr_6_/Ti_3_C_2_	RhB in ethanol	100% in 70 min	[38]
7	NGQDs-CsPbI_3_/TiO_2_	RhB in water	96% in 4 h	our

## Data Availability

The data can be made available upon reasonable request.

## Supplementary Materials

The following supporting information can be downloaded at: https://www.mdpi.com/article/10.3390/molecules28217310/s1, Figure S1. (a) TEM image of NGQDs. (b) Statistical size distribution of the prepared NGQDs. (c) XPS full spectrum of NGQDs. (d) High-resolution of N 1s and C 1s spectra of NGQDs. Figure S2. (a) TEM images of δ-phase CsPbI_3_ nanocrystals. (b) Statistical size distribution of the prepared δ-phase CsPbI_3_. (c) and (d) High-resolution TEM images of NGQDs-CsPbI_3_ with different space stripes. Figure S3. (a) EDS mapping and (b) the atomic proportion of NGQDs-CsPbI_3_. Figure S4. Comparison of the photodegradation ratios of RhB obtained by the absorbance (Abs) and total organic carbon (TOC) analysis of RhB solution. Figure S5. The RhB photodegradation activities of different NGQDs-CsPbI_3_/TiO_2_ samples by tuning the initial NGQDs mass. The NGQDs mass is 0.5, 0.7 and 0.9 mg in NGQDs-1-CsPbI_3_, NGQDs-CsPbI_3_ and NGQDs-2-CsPbI_3_, respectively, and the CsPbI_3_ part is 72 mg. The mass of TiO_2_ is 250 mg. Table S1. Quantum yields of δ-CsPbI_3_, NGQDs, NGQDs-CsPbI_3_ and NGQDs-CsPbI_3_/TiO_2_ using quinine sulfate as a reference.
