# Supplementary material for: Nitrogen-Doped Graphene Quantum Dot-Passivated δ-Phase CsPbI3: A Water-Stable Photocatalytic Adjuvant to Degrade Rhodamine B
Source: Molecules. 2023 Oct 28;28(21):7310. doi: 10.3390/molecules28217310 (PMC10650061; doi:10.3390/molecules28217310)
Supplement: Supplementary file 1 [file molecules-28-07310-s001.zip › molecules-2655629-supplementary.pdf]

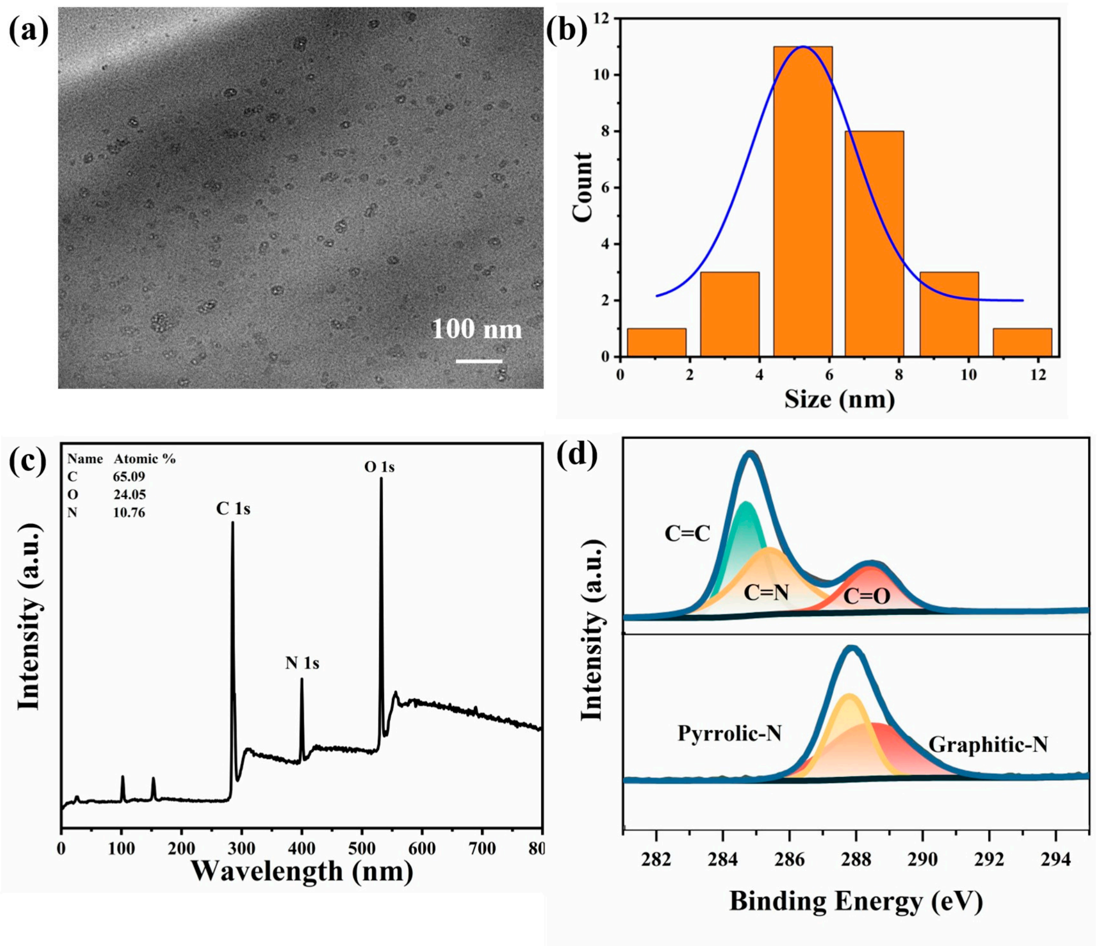

Figure S1. (**a**) TEM image of NGQDs. (**b**) Statistical size distribution of the prepared NGQDs. (**c**) XPS full spectrum of NGQDs. (**d**) High-resolution of N 1s and C 1s spectra of NGQDs.

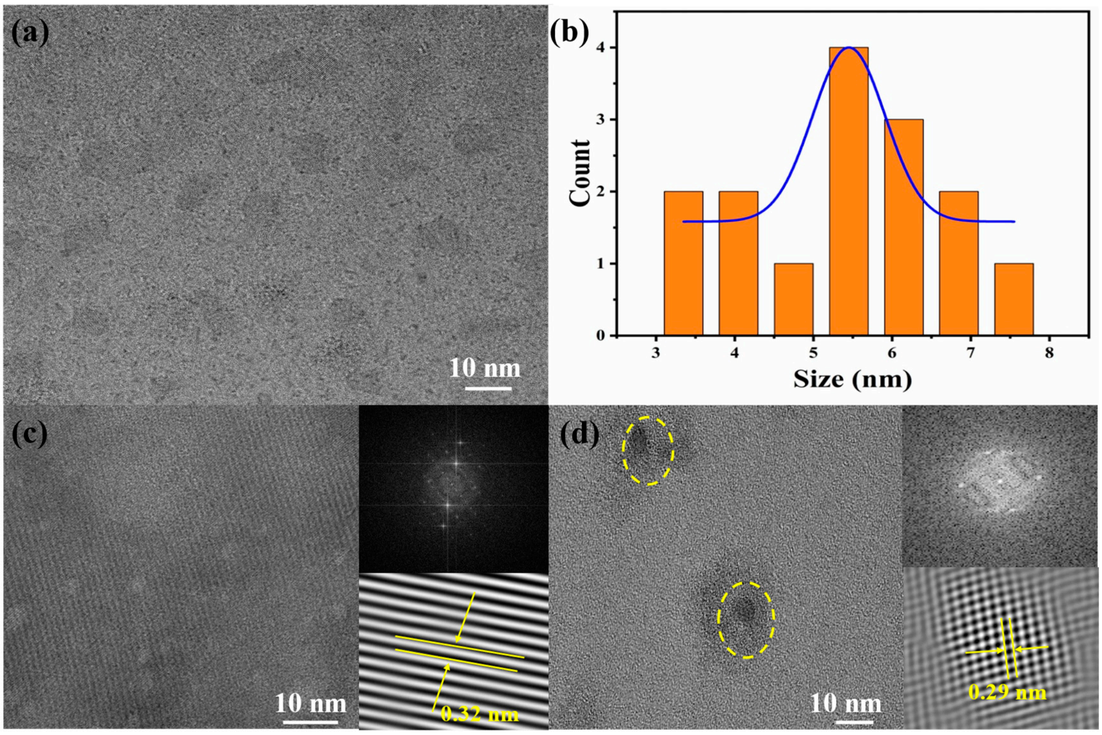

Figure S2. (**a**) TEM images of δ-phase $CsPbI_3$ nanocrystals. (**b**) Statistical size distribution of the prepared δ-phase $CsPbI_3$. (**c**) and (**d**) High-resolution TEM images of NGQDs-$CsPbI_3$ with different space stripes.

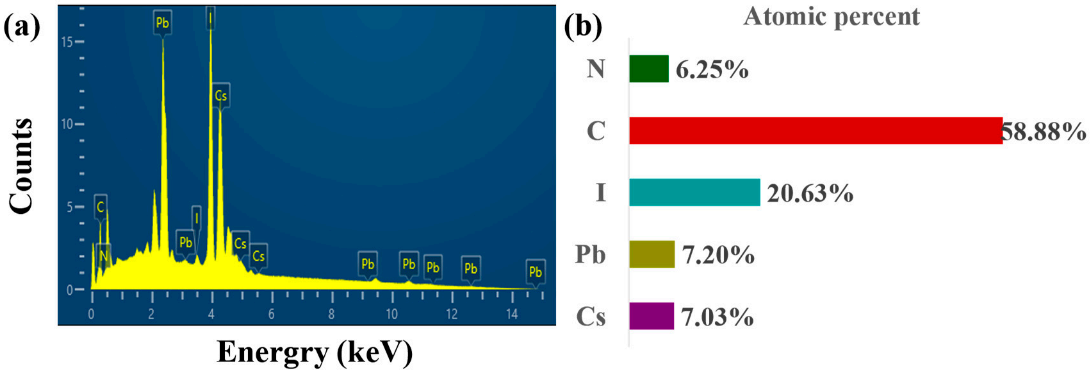

Figure S3. (**a**) EDS mapping and (**b**) the atomic proportion of NGQDs-$CsPbI_3$.

Table S1. Quantum yields of δ-$CsPbI_3$, NGQDs, NGQDs-$CsPbI_3$ and NGQDs-$CsPbI_3/TiO_2$ using quinine sulfate as a reference.

Sample	Integrated emission intensity (I)	Abs. (A)	Refractive index of solvent (n)	Quantum yield (Φ)
Quinine sulfate	22615.06	0.08	1.33	0.54 (known)
δ-$CsPbI_3$	2069.04	1.09	1.33	0.0036
NGQDs	16007.34	1.09	1.33	0.0281
NGQDs-$CsPbI_3$	19008.36	1.09	1.33	0.0333
NGQDs-$CsPbI_3/TiO_2$	11588.55	1.09	1.33	0.0203

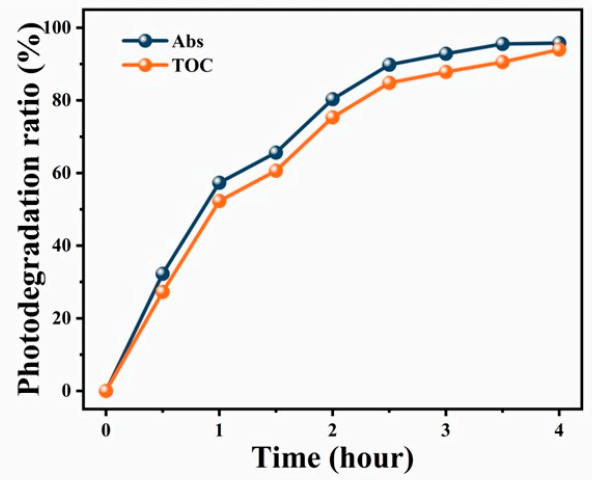

Figure S4. Comparison of the photodegradation ratios of RhB obtained by the absorbance (Abs) and total organic carbon (TOC) analysis of RhB solution.

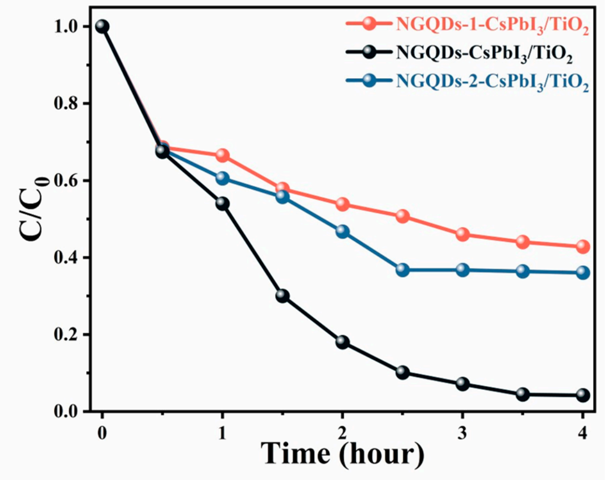

Figure S5. The RhB photodegradation activities of different NGQDs-$CsPbI_3/TiO_2$ samples by tuning the initial NGQDs mass. The NGQDs mass is 0.5, 0.7 and 0.9 mg in NGQDs-1-$CsPbI_3$, NGQDs-$CsPbI_3$ and NGQDs-2-$CsPbI_3$, respectively, and the $CsPbI_3$ part is 72 mg. The mass of TiO_2 is 250 mg.
